# Effect of NaCl on the Lifetime of Micro- and Nanobubbles

**DOI:** 10.3390/nano6020031

**Published:** 2016-02-05

**Authors:** Tsutomu Uchida, Shu Liu, Masatoshi Enari, Seiichi Oshita, Kenji Yamazaki, Kazutoshi Gohara

**Affiliations:** 1 Division of Applied Physics, Faculty of Engineering, Hokkaido University, N13 W8 Kita-ku, Sapporo, Hokkaido 060-8628, Japan; k-yamazaki@eng.hokudai.ac.jp (K.Y.); gohara@eng.hokudai.ac.jp (K.G.); 2 Graduate School of Agricultural & Life Sciences, The University of Tokyo, Yayoi 1-1-1, Bunkyo-ku, Tokyo 113-8657, Japan; liu@bpe.en.a.u-tokyo.ac.jp (S.L.); masatoshi.enari@gmail.com (M.E.); aoshita@mail.ecc.u-tokyo.ac.jp (S.O.)

**Keywords:** microbubble, nanobubble, freeze-fracture replica, transmission electron microscope, ζ-potential, ionic shielding, diffusive shielding

## Abstract

Micro- and nanobubbles (MNBs) are potentially useful for industrial applications such as the purification of wastewater and the promotion of physiological activities of living organisms. To develop such applications, we should understand their properties and behavior, such as their lifetime and their number density in solution. In the present study, we observed oxygen MNBs distributed in an electrolyte (NaCl) solution using a transmission electron microscope to analyze samples made with the freeze-fracture replica method. We found that MNBs in a 100 mM NaCl solution remain for at least 1 week, but at higher concentrations decay more quickly. To better understand their lifetimes, we compared measurements of the solution's dissolved oxygen concentration and the ζ-potential of the MNBs. Our detailed observations of transmission electron microscopy (TEM) images allows us to conclude that low concentrations of NaCl stabilize MNBs due to the ion shielding effect. However, higher concentrations accelerate their disappearance by reducing the repulsive force between MNBs.

## 1. Introduction

Small gas bubbles have great potential in industrial applications such as the purification of wastewater, water-quality improvement, sterilization, decolorization, and the promotion of physiological activities of living organisms [1]. These bubbles include micrometer-scale bubbles, or microbubbles (MBs), as well as nanometer-scale bubbles, or nanobubbles (NBs). As there is no consensus about the exact distinction between these two scales, we lump them together here, calling them “micro- and nanobubbles (MNBs)”.

MNBs have unique properties that make them suitable for such applications. These properties include a large specific area and, as estimated from the Young-Laplace equation, a high gas pressure. Furthermore, MNBs can have an electrically charged surface [2,3,4]. One study of the ζ-potential shows the surface charge of MNBs to be negative over a wide range of pH, and attributes this charge to a dominance of hydroxide ions in the first molecular layers of water at the gas-liquid interface [5]. Such charging would inhibit the coalescence of MNBs in water.

Regarding fluid dynamic properties, MNBs in water have a small rising speed and act to reduce the frictional resistance [6]. According to Stoke’s law, a smaller bubble has a smaller rising speed and thus tends to stay longer at a specific area in fluid. Such behavior may explain the observed promotion of physiological activity and sterilization effect of water containing MNBs [1,7,8].

However, the lifetime of MNBs remains unclear. For example, a quasi-stationary model suggests that a MB in pure water dissolves in less than 1 s [9]. Another estimate [10] for air bubbles of radii 10–100 nm gives a lifetime of 1–100 ns. But the lifetime can be increased to hundreds of seconds [11] or even to over a few months [12] by the addition of surfactant molecules that lower the interfacial tension and decrease the pressure difference between inner bubble and surroundings. Thus, surfactants may be useful in industrial applications of MNB, but may also increase environmental concerns.

The effect of electrolytes on MNB lifetime is more complicated. A small amount of electrolyte is added to “stabilize” MNB in a commercially available solution [7]. Seddon *et al.* [13] suggested that the electrolyte increases the lifetime of dispersed NBs (“bulk NBs”) due to ionic shielding and diffusive shielding effects. Zheng *et al.* [14], on the other hand, investigated the morphology of NBs fixed on a solid surface (“surface NBs”) in various solutions containing salts, acids and bases. They found that the surface NBs, once formed, were insensitive to the addition of salts, and to the pH of the aqueous phase. However, Takahashi [2] found that the increase in the concentration of electrolytes (NaCl and MgCl_2_) decreases (*i.e.*, makes less negative) the ζ-potential of MBs.

Another parameter related to MNB lifetime is the structure of the surrounding water. For example, hydrophobic gas molecules can create a highly ordered, ice-like hydration shell [15], and high-charge-density ions can act as “structure-makers” in aqueous solution [16]. These mechanisms are, however, still not universally accepted [17].

Understanding the effect of electrolytes on the MNB lifetime is important not only for the fundamental knowledge of the origin of the surface charge of MNBs, but also for the industrial applications of MNBs in various conditions, such as the promotion of physiological activities of living organisms in the culture medium or in sea water.

All studies mentioned above highlight a considerable uncertainty of MNB behavior and properties in practical solutions. This situation is mainly due to MNBs being hard to observe directly, especially NBs, which are below optical resolution and flow in fluid. Although surface MNBs have been observed directly by atomic force microscopy (AFM) [14,18,19], they likely have different physico-chemical properties than bulk NBs [13], and we do not have such a tool for bulk NBs yet. For non-optical-microscope studies of bulk MNBs, we have used dynamic light scattering (DLS) and particle tracking analysis [3,8]. In another method, Uchida *et al.* [20] applied transmission electron microscopy (TEM) to freeze-fracture replicas of bulk MNBs, and found that bulk NBs collected impurities in the solution on the MNB surface, a potential purification process of wastewater.

Here, we used the same TEM–freeze-fracture replica method to observe bulk oxygen (O_2_) MNB distributions in various concentrations of NaCl solutions (from pure water to 3.5 wt % NaCl). The purpose was to investigate the effect of electrolytes on the lifetime of O_2_-MNBs. In particular, we analyzed how the O_2_-MNB distribution changes in storage for up to one week at room temperature. To better understand the mechanism through which electrolytes affect the O_2_-MNB distribution, we also measured pH and the amount of dissolved O_2_ (DO) of the NaCl–MNB solutions as well as the ζ-potential of the O_2_-MNB.

## 2. Experimental Section

To prepare the solutions, NaCl (high purity, Kanto Chem., Tokyo, Japan) was dissolved in 1 L of ultra-high purity water (Kanto Chem., Japan). This solution and pure O_2_ gas (purity 99.99995%, Taiyo Nippon Sanso, Kanagawa, Japan) were introduced into a MNB generator (Aura Tech, Fukuoka, Japan, type OM4-GP-040) at a temperature of about 293 K and a pressure of 0.26 ± 0.01 MPa. Under the above conditions, the flow rate of O_2_ gas was about 0.1 NL·min^−1^ and the capacity of the MNB generator about 0.95 L·min^−1^. After 1 h of bubble generation, which allows the MNB number to stabilize [21], the solution was stored in a sealed glass sample bottle at 293 K.

The details of the replica sample preparation have been described in the previous work [22], so here we explain them briefly. A small amount of solution (10–20 mm^3^) is removed from the stored bottle after the designated time, put on an Au-coated Cu sample holder, and then rapidly plunged into liquid nitrogen. In this condition, the freezing rate is of order 10^3^ K·min^−1^. Then, the frozen droplet is fractured under vacuum (10^−4^–10^−5^ Pa) and low temperature (~100 K) to reduce artifact formation. A replica film of this fractured surface is prepared by evaporating platinum and carbon on it (JEOL, Tokyo, Japan, type JFD-9010).

After the sample is taken out from the freeze-fracture replica unit, the replica film is removed from the ice body by melting it in pure water at room temperature. The replica film is then transferred to the Cu-fine grid (Nisshin EM, Tokyo, Japan, type F-400), which has a 43 μm^2^ open mesh. We use a field-emission gun-type TEM at 200 kV (JEOL, type JEM-2010F, Tokyo, Japan) to observe the replica film. An imaging plate (Fujifilm, Tokyo, Japan, type FDL-UR-V) is used to acquire the observed image. We observed the morphologies of MNBs, counted the number of MNBs in a square hole, and measured the MNB size (both major and minor axis of the oval hole) as an equivalent spherical diameter having the same cross-section area. As the diameter distribution is not simple, the average MNB diameter *D* is calculated as the arithmetic average of total number of measured MNBs (*n*). For averaging the diameter and number of MNBs, we measure at least 30 MNBs for each solution sample (except in the 3.5 wt % NaCl solution). Then, to estimate the MNB number density *N* mL^−1^, we assume a uniform MNB distribution and use

*N*^2^ = (<*n*>/s)^3^ (1)

where <*n*> is the average number of MNBs per one open square hole having the area *s* (1.85 × 10^−5^ cm^2^). The uncertainty of *N* is estimated as the standard error of <*n*>, although the distribution is not fully fitted to a normal distribution.

The DO concentration and pH are measured simultaneously in the NaCl–MNB solution. The DO concentration is determined using a DO meter (Mettler-Toledo, Schwerzenbach, Switzerland, type SG6) with an accuracy of ±0.5%. All measurements are done at a controlled temperature of 293 K. The pH is measured with a pH meter (Horiba, Kyoto, Japan, type D-55), which has a range of 0 to 14 with accuracy ±0.01. The pH meter automatically compensates the pH values for 293 K ± 2 K.

For the ζ-potential measurements, we use a Zeta Potential Analyzer (Microtech, Chiba, Japan, type Zeecom ZC-3000). This system, equipped with a microscope and a CCD camera, is used to observe the electrophoretic mobility of MNBs in the range of 20 nm to 100 μm. The small MNBs can be observed by the scattering of halogen light. The sample was measured in a glass cell of size 1 mm × 10 mm. The sample volume was 8 mL and the distance between the electrodes was 9 cm. The voltage was set to 30 V DC. Fifty MNBs were manually tracked to measure their speed and the ζ-potential was then estimated by the Smoluchowski equation [3,4]. Due to the dielectric influence from NaCl, we could not measure the ζ-potential of MNBs in NaCl solutions at a concentration exceeding 100 mM, at least in the present experimental system.

## 3. Results and Discussion

To determine how the MNB distribution depends on the NaCl concentration, we prepared NaCl–MNB solutions with NaCl concentrations of 0 mM, 10 mM, 100 mM, 1 wt % (173 mM), and 3.5 wt % (605 mM). Then, we stored the sample solutions for a fixed period of up to 7 days at 293 K.

**Figure 1:**
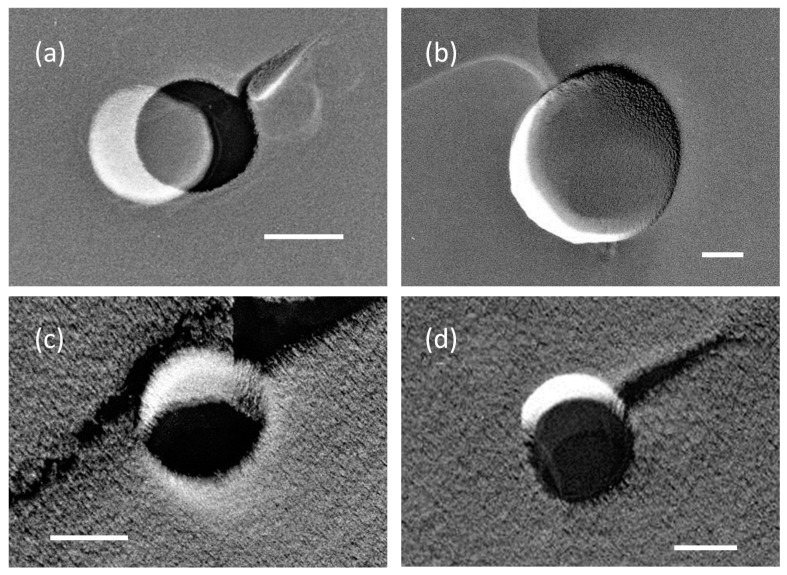
Transmission electron microscopy (TEM) images of micro- and nanobubbles (MNBs) in (**a**,**b**) 10 mM and (**c**,**d**) 100 mM NaCl solutions. Each scale bar shows 100 nm. A bubble hole was sometimes covered with thin film (relatively brighter image) formed inside of the hole.

**Figure 2:**
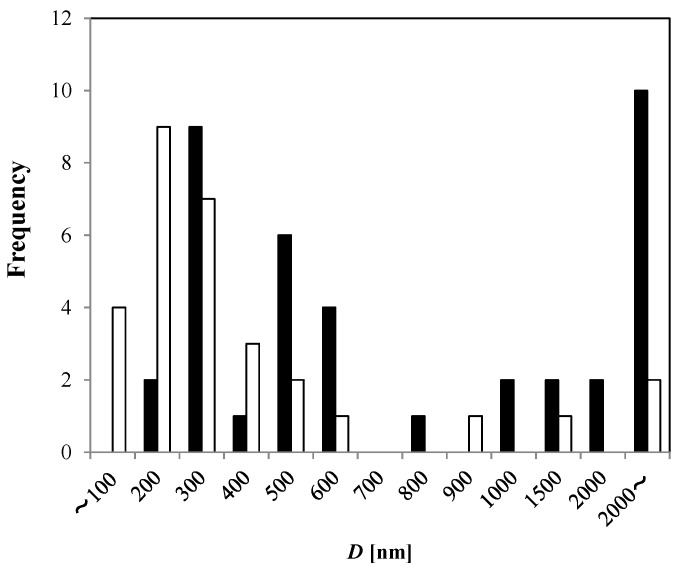
Apparent bubble diameter (*D*) observed from TEM-freeze-fracture replica method in 0 mM NaCl solution after 1 h MNB generation (solid bar, *n* = 39), and in pure water without MNB generation (open bar, *n* = 31).

The replica samples showed circular or oval-shaped holes on the freeze-fractured cross-section with apparent diameters from several tens of nanometers to several microns. See Figure 1 for several examples. From such TEM images, we then measured the MNB size distribution. MNBs were also found in the pure water sample that did not undergo MNB generation. Such bubbles would have been generated during sample freezing due to the rapid decrease of gas solubility in solution with a temperature decrease. Figure 2 shows how the bubble distribution changed after 1 h of MNB generation.

Compared to the pure water without MNB generation, the generated MNBs in 0 mM NaCl solution (1 h bubble generation with pure water) ranged in sizes between 200 and 600 nm and larger than 2 μm. The number density for the 0 mM NaCl solution was *N_0_* = (5.1 ± 0.3) × 10^8^·mL^−1^, and that for the pure water without MNB generation is *N_w_* = (4.5 ± 4.9) × 10^7^·mL^−1^. (Although the distribution did not fully fit to a normal distribution, we used the normal error here for the uncertainties.) Therefore, according to this freeze-fracture method, the generator increased the total number density of MNBs by a factor of ten.

**Figure 3:**
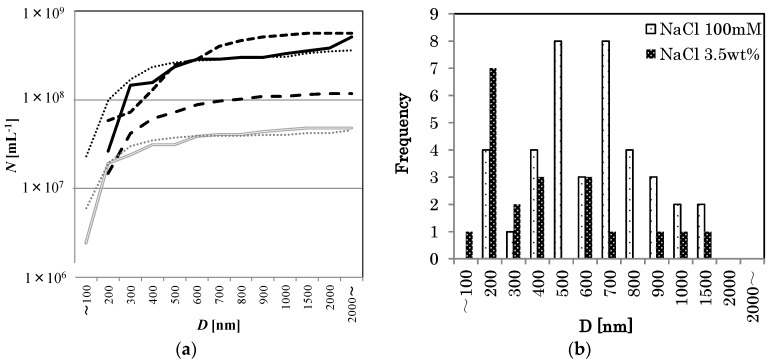
(**a**) The cumulative distributions of *D* in different NaCl concentrations (0 mM: Solid line, 10 mM: larger dashed line, 100 mM: Smaller dashed line, 1 wt %: Dotted line, 3.5 wt %: Double solid line, pure water: thin dotted line). The value at the largest *D* equals the number density *N*. (**b**) Apparent bubble diameter distributions in 100 mM NaCl solution (coarse dot bar, *n* = 39), and in 3.5 wt % NaCl solution (fine dot bar, *n* = 20).

Consider now the effect of NaCl concentration on the O_2_-MNB distribution. In all samples with NaCl, the generated MNBs had a larger bubble number density than *N_w_*. See Figure 3 for the cumulative distributions of *D* in these different NaCl concentrations. In the figure, the maximum value of each line equals the total number density. The results also show that the bubble diameter distribution in weaker NaCl solutions (less than about 500 mM) forms a peak near several hundred nanometers and that *N* exceeds 10^8^·mL^−1^ (for example, 100 mM NaCl solution: smaller dashed line in Figure 3a and coarse dot bar in Figure 3b). However, in stronger NaCl solutions (for example, 3.5 wt % NaCl solution: double solid line in Figure 3a and fine dot bar in Figure 3b), *N* tends to decrease to about 10^7^·mL^−1^, which is the same order of magnitude as that of the pure water without MNB generation.

**Figure 4:**
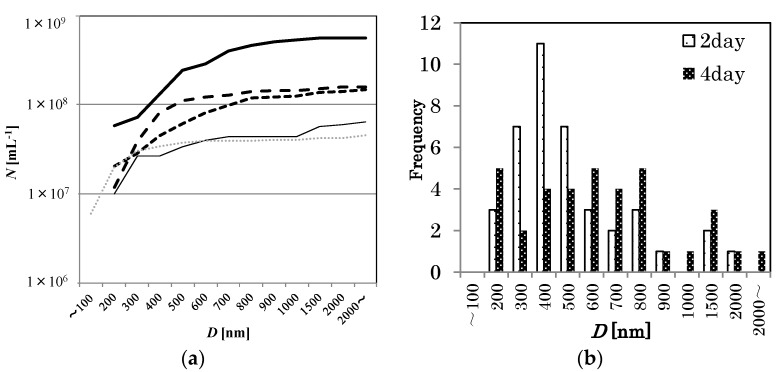
(**a**) Similar to Figure 3 except for 100 mM NaCl solution at various storage periods at 293 K (0 day: thick solid line, 2 days: larger dashed line, 4 days: smaller dashed line, 7 days: thin solid line, pure water: thin dotted line). (**b**) Apparent bubble diameter distributions in 100 mM NaCl solution 2 days after MNB generation (coarse dot bar, *n* = 40), and 4 days after MNB generation (fine dot bar, *n* = 36).

**Figure 5:**
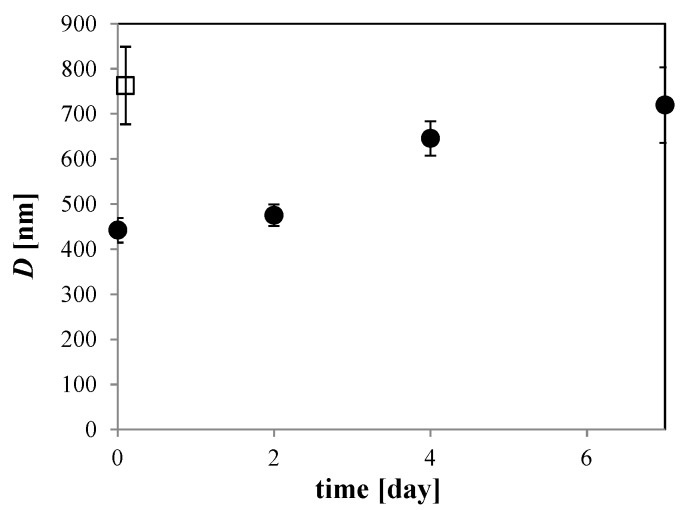
Storage time dependence of *D* in 100 mM NaCl solution at 293 K (solid circle). Open squares show *D* in pure water. Error bars are the standard error based on a normal distribution.

The distribution change of O_2_ MNBs that were sealed in glass bottles was investigated for a 100 mM NaCl solution. See Figure 4 for the changes in distribution. At least for this temperature (293 K), the number of micrometer-scale bubbles first decreased (for example, compare 0 day in Figure 2 solid bar and 2 days in Figure 4b coarse dot bar), resulting in a single-peak distribution, and the number of smaller bubbles then started to decrease (see Figure 4a,b), causing the average diameter to increase. After 7 days, the distribution of MNBs resembled that of the control (*i.e.*, pure water without bubble generation). *D* has a minimum just after bubble generation, and then increases with time (Figure 5). Thus, these results suggest that O_2_ MNBs in NaCl solutions increase in average diameter as their numbers decrease.

**Figure 6:**
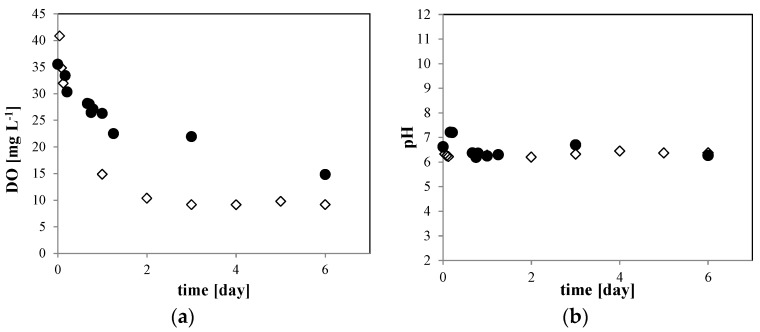
Time dependence of (**a**) DO concentration and (**b**) pH of the O_2_ MNB in the solution including 0 mM (open diamond) and 10 mM NaCl (solid circle). The measurement uncertainties are within the size of each symbol.

Now consider the 10 mM NaCl solution. In Figure 6, we compare (a) the dissolved O_2_ (DO) concentration and (b) the pH of the O_2_ MNB for the 10 mM NaCl solution to that for 0 mM NaCl solution with bubble generation. In the 0 mM NaCl solution, the DO concentration (Figure 6a, open diamond) changed from about 45 mg·L^−1^ just after the MNB generation to about 10 mg·L^−1^ at 6 days after generation, which approaches the O_2_ saturation equilibrium. In the 10 mM NaCl solution (Figure 6a, solid circle), the DO concentration decreased slightly more slowly than that in the 0 mM NaCl solution. The pH (Figure 6b) remained at about 6.2 in both solutions during the storage period. These results indicate that the excess dissolved O_2_ gradually diffuses out of the aqueous phase to the atmosphere, but the concentration stays above the supersaturation level during the storage period. In agreement with previous studies [3,4], we found that the O_2_ MNBs is the source of O_2_ molecules in the liquid phase.

**Figure 7:**
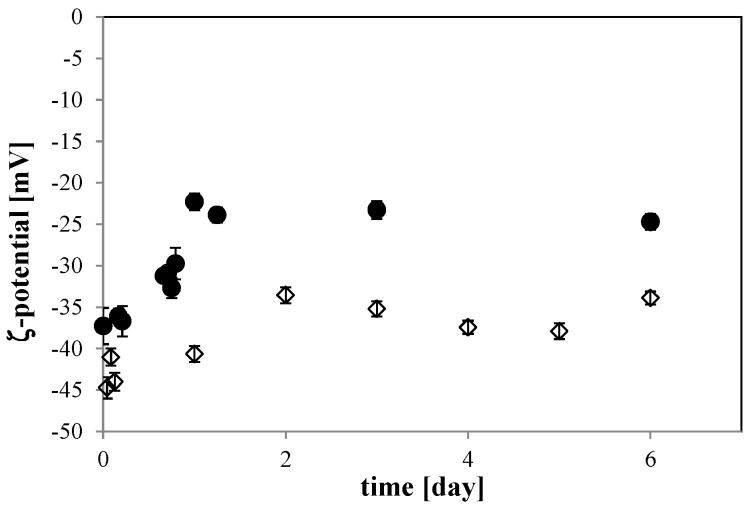
Time dependence of the ζ-potential of the O_2_ MNB solution including 10 mM NaCl (solid circle) and no additives (open diamond). Each error bar shows the standard deviation of the measurement.

For the 10 mM NaCl solution, the ζ-potential of the O_2_ MNB changed with time. It went from about −40 mV just after MNB generation to about −25 mV during the first day of storage, then became constant until 6 days of storage (solid circle in Figure 7). This variation is relatively rapid, being larger than that of O_2_ MNB in the 0 mM solution (open diamond in Figure 7), which changed from −45 to −35 mV during 2 days of storage. In this solution, the ζ-potential variation is similar to that in previous studies [3,4]. Another previous study measured the ζ-potential of air MNBs in a 10 mM NaCl solution, finding a value that varied with pH, being about −20 mV at a pH of about 6.2 [23]. Our finding of a larger absolute ζ-potential is consistent with earlier studies that found a smaller ζ-potential for air MNB than that for O_2_ MNB [3,4]. Here, the pH was nearly constant, so our finding a difference in ζ-potential for O_2_ MNBs is likely due to the effect of NaCl.

According to other studies [3,4,24], a high absolute ζ-potential stabilizes the bubble distribution through repulsion, which reduces bubble coalescence. The high absolute ζ-potential value of O_2_ MNBs in the 0 mM NaCl solution observed in the present study would have prevented the bubble coalescence, and it is considered to be determined by the amount of ions (H^+^ and OH^−^) and their valences in the slipping plane [2]. By adding NaCl to the solution, positive ions (mainly Na^+^) would have been attracted to the bubble surface. Since the pH value was almost constant, the accumulation of positive ions near the bubble surface would have decreased the absolute ζ-potential. Then, the ζ-potential of MNBs in a high NaCl concentration solution would have been smaller, going below the critical value that can inhibit MNB coalescence.

This is also considered by an estimate of the Debye length *λ_D_*, the radius in which the excess ionic charge is approximately equal to charge opposite value of the MNB:

(2) $$\lambda_{D}=\sqrt{\left(\frac{\varepsilon_{r}\varepsilon_{0}kT}{\sum_{i}z_{i}e^{2}n_{i}}\right)}$$

where *ε*_r_ and *ε*_0_ are the specific permittivity of the solution and the dielectric constant in the vacuum, respectively, *k* is Boltzmann's constant, *T* is the absolute temperature, *z_i_* is the ion valance, *e* is the electronic charge, and *n_i_* is the ion concentration in the solution [25]. For the NaCl solution at 298 K, Equation (1) yields *λ_D_* ~0.304 [NaCl]^−1/2^, where [NaCl] is the NaCl concentration [25]. If we assume that our ’’pure water’’ has [NaCl] ~10^−7^ M at 298 K, then this water would have a *λ_D_* of about 10^3^ nm. For this water, adding 10 mM NaCl would have reduced *λ_D_* by a factor of 10^−3^ (to about 3 nm). For a larger NaCl concentration, *λ_D_* would have become even smaller.

Such a decrease in *λ_D_* is related to the decrease of the absolute ζ-potential, which would have reduced the repulsive force between bubbles, increasing the rate of bubble coalescence, thus causing a rapid decrease in *N*. This phenomenon was observed in the 100 mM NaCl solution at an early stage of the storage period (within 2 days, Figure 4). If the NaCl concentration was high enough, the lifetime of MNBs would be short. Thus, the NaCl concentration dependence of MNB distribution (Figure 3) can be explained by this mechanism. The surface NBs are, on the other hand, insensitive to the addition of electrolytes [14] because they are fixed on the solid surface and their coalescent effect would be negligible.

However, after two days of storage, both the DO value (Figure 6a) and the ζ-potential (Figure 7) became constant, whereas *N* gradually decreased while *D* increased, as shown in Figure 4 and Figure 5. During this stage, the number of smaller MNBs tended to decrease (Figure 4). These smaller bubbles should have had a larger inner pressure, in particular, according to the Young-Laplace equation

*ΔP* = 2*σr*^−1^ (3)

where *ΔP* is the pressure difference across the bubble surface, *σ* is the interfacial tension, and *r* is the bubble radius. According to Henry’s law and the Gibbs-Thomson equation, the smaller sized bubbles have a larger potential as a source of O_2_ into the surrounding solution. Thus, the MNB distribution change should be controlled by the diffusion of O_2_ from smaller bubbles to larger bubbles via Ostwald ripening, or to the atmosphere through the O_2_-saturated liquid phase.

**Figure 8:**
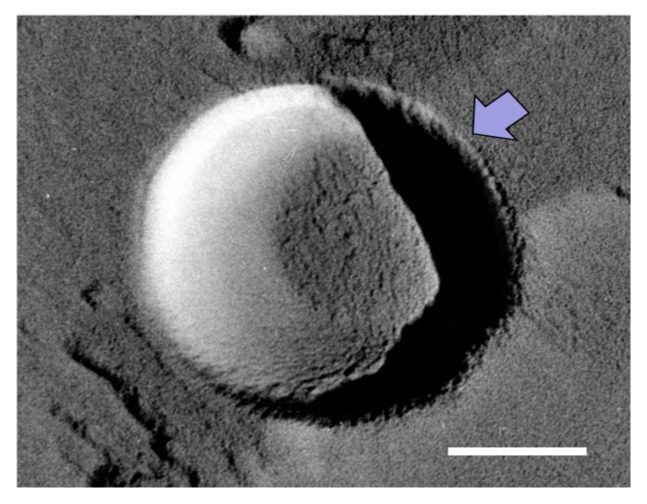
TEM image of a MNB in 1 wt % NaCl solution. Scale bar shows 100 nm. Arrow marks the thin layer on the MNB.

We shall now consider this “semi-stable” state in the O_2_ MNB–NaCl solution. If the dissolution rate of O_2_ from a MNB into the surrounding solution is large enough, the lifetime of the MNB should theoretically be very short [10]. However, in an actual solution, the dissolution rate would be reduced by a diffusion barrier, which could come from an accumulation of anions or cations [13] (ionic shielding), an accumulation of surfactant [12], or from a highly structured hydrogen-bonding network [15]. In our TEM observations, we sometimes detect a thin layer (less than 10 nm thick) on the NB surface (see the arrow in Figure 8). This layer was frequently observed in solutions with a higher NaCl concentration. Previously, Uchida *et al.* [20] observed the freeze-fracture replica of a NaCl solution, which showed that some impurities precipitated in the boundary between ice crystals. Although we could not determine what formed the thin layer on NBs, due to the observed sample being a replica formed by platinum and carbon film, we speculate that this thin layer was the precipitation of impurities (including NaCl and NaCl–2H_2_O). In the aqueous phase, Na^+^ should accumulate near the slipping plane, as mentioned above. Thus, this ion-accumulation zone reduces the dissolution rate of O_2_ from the bubble to the surrounding solution (via ionic shielding).

In addition to the ionic shielding effect, it is important for the “semi-stable” state of MNBs to maintain the DO concentration above O_2_ saturation. In the present study, the DO concentration remained above O_2_ saturation for at least 1 week in the 0 mM NaCl–MNB solution. Some previous works [13,26] have shown that the lifetime of MNBs depends on the dissolved gas concentration, and thus on *N*. If enough MNBs (source) exist in the solution, the solution remains at the saturation level, a condition that would reduce the dissolution rate of gas from MNBs (diffusive shielding). In our experiments, the MNB solutions were stored in a sealed glass bottle at a constant temperature. Thus, the evaporation of O_2_ from liquid to atmosphere was likely suppressed enough to keep the saturation condition for a week. Although under such a condition, *N* should decrease continuously with a gradual release of O_2_ from the liquid phase. When *N* falls below the lower limit for maintaining gas saturation, the MNBs should disappear quicker than before.

Therefore, we argue that a small amount of NaCl in solution helps maintain the MNBs in the solution via the ionic shielding effect. The TEM images show the existence of an ion-accumulation zone on NBs. The sealed bottle also plays an important role in the stability of bulk MNBs in the solution via the diffusive shielding effect. These results support the generally accepted idea that a small amount of electrolyte stabilizes bulk MNBs. On the other hand, a large amount shortens the MNB lifetime by accelerating the coalescence of MNBs via a decrease in both *λ_D_* and the MNB ζ-potential. This is a disadvantage for the industrial applications of MNBs in the solution with a larger electrolyte concentration, such as in sea water. In such a case, MNBs cannot stay longer in the solution. Thus, a continuous supply of MNBs in the system would be necessary to use them as a functional material.

## 4. Conclusions

We used a TEM–freeze-fracture replica method to observe the distribution of micro- and nanobubbles of oxygen (O_2_–MNBs) in solutions of NaCl concentration below 3.5 wt % at 293 K. We demonstrated that this observation technique is valuable for direct observation of MNBs, not only in pure water, but also in electrolyte solutions. The distribution of O_2_-MNBs depended on both the NaCl concentration and the storage period. The MNB number concentration was smaller at higher NaCl concentrations, and gradually decreased at longer storage periods. Using the ζ-potential of the O_2_-MNB and dissolved O_2_ (DO) concentrations in the solution, we analyzed the effect of NaCl on the MNB distributions. Based on TEM images that show the thin layer formation on O_2_ MNBs, we argue that a small amount of NaCl in solution helps to maintain the MNBs via the ionic shielding effect. However, a larger amount of NaCl shortens the lifetime of MNBs by accelerating MNB coalescence due to a decrease in both the Debye length and the ζ-potential of an O_2_ MNB.
